# The Links Between the Gut Microbiome, Aging, Modern Lifestyle and Alzheimer's Disease

**DOI:** 10.3389/fcimb.2020.00104

**Published:** 2020-03-18

**Authors:** Sholpan Askarova, Bauyrzhan Umbayev, Abdul-Razak Masoud, Aiym Kaiyrlykyzy, Yuliya Safarova, Andrey Tsoy, Farkhad Olzhayev, Almagul Kushugulova

**Affiliations:** National Laboratory Astana, Center for Life Sciences, Nazarbayev University, Nur-Sultan, Kazakhstan

**Keywords:** Alzheimer's disease, gut microbiome, aging, lifestyle, circadian rhythm

## Abstract

Gut microbiome is a community of microorganisms in the gastrointestinal tract. These bacteria have a tremendous impact on the human physiology in healthy individuals and during an illness. Intestinal microbiome can influence one's health either directly by secreting biologically active substances such as vitamins, essential amino acids, lipids et cetera or indirectly by modulating metabolic processes and the immune system. In recent years considerable information has been accumulated on the relationship between gut microbiome and brain functions. Moreover, significant quantitative and qualitative changes of gut microbiome have been reported in patients with Alzheimer's disease. On the other hand, gut microbiome is highly sensitive to negative external lifestyle aspects, such as diet, sleep deprivation, circadian rhythm disturbance, chronic noise, and sedentary behavior, which are also considered as important risk factors for the development of sporadic Alzheimer's disease. In this regard, this review is focused on analyzing the links between gut microbiome, modern lifestyle, aging, and Alzheimer's disease.

## Introduction

Alzheimer's disease (AD) is a progressive neurodegenerative disease characterized by memory loss, dramatic changes in character and behavior and an impossibility to carry out normal daily activities in the latter stages of the disease. AD incidence increases with age and is shown to affect ~10% of people aged 65–75 and 32% of the elderly aged 80 and above (Alzheimer's Association, 2013; Prince et al., 2016). According to the World Health Organization (WHO) incidence of AD is worsening every year, thus it is postulated that there could be a threefold increase in the number of AD patients by 2050. Today it is believed that the pathophysiology of AD is driven by accumulation of different forms of amyloid beta peptide (Aß) in the brain leading to neuro-inflammation, oxidative stress, mitochondrial dysfunction, dysregulation of enzyme systems, and neuronal death. Yet, triggering mechanisms of Aβ deposition in the brain are still being investigated. To date, <5% of all AD cases have clear genetic evidence of increased production of Aβ. Mutations in three genes serve to transmit AD via autosomal-dominant inheritance: the presenilin gene (PS1) on chromosome 14, the presenilin 2 gene (PS2) on chromosome 1, and the amyloid precursor protein gene (APP) on chromosome 21. This form of AD is referred to as a familial Alzheimer's disease (FAD) and is characterized by earlier onset of the symptoms (EOAD, <65 years). However, most cases of AD have a late-onset of the symptoms (≥65 years) and an unclear genetic background (Panegyres and Chen, 2013, 2014). This type of dementia is called sporadic late-onset AD (LOAD), and it is generally believed that the development of this pathology in the elderly is as a result of an interplay between genetic background and various factors including lifestyle, stress levels, chronic diseases (cardiovascular diseases, obesity, diabetes mellitus), and environment (Prince et al., 2014; Wainaina et al., 2014).

One of the important factors that is influencing human health and attracting increasing attention of scientists during the last two decades is gut microbiome. There are ~1,000 species and 7,000 strains of bacteria that inhabit the human intestine (1,013–1,014 microorganisms in total), among which the most common are bacteria attributed to *Firmicutes* (51%) and *Bacteroidetes* (48%) (The Human Microbiome Project et al., 2012). *Firmicutes* include both gram-positive and gram-negative species such as those belonging to the genus *Lactobacillus* (gram-positive), *Eubacterium* (gram-positive), *Clostridium* (gram-positive). *Bacteroidetes* comprise of gram-negative bacteria of the genus *Bacteroides* and *Prevotella*. The remaining 1% of bacteria belong to other divisions such as *Proteobacteria* (gram-negative, genus *Escherichia* in particular), *Actinobacteria* (gram-positive, genus *Bifidobacterium* in particular), *Fusobacteria* (gram-negative), *Spirochaetes* (gram-negative), *Verrucomicrobia* (gram-negative), and *Lentispherae* (gram-negative) (Westfall et al., 2017). Until recently, intestinal microbiome was considered to be involved in processes that take place exclusively in the intestine, such as fermentation of carbohydrates, synthesis of vitamins (in particular vitamin B and K), and xenobiotic metabolism as well as acting as a barrier to pathological bacteria. However, over the last 15 years, the functions of the intestinal microbiome have been revised owing to the establishment of a direct link between density and species composition of the intestinal microbiome and a number of pathological conditions including diabetes, obesity, and cardiovascular diseases. These diseases, in turn, are the established risk factors for the development of sporadic AD, and there is data indicating that gut microbiome influences brain functions (Westfall et al., 2017; Zhu et al., 2017; Kowalski and Mulak, 2019). Moreover, recent studies have revealed the significant differences in quantity and quality of gut microbiome in AD patients compared to mentally healthy individuals of the same age (Vogt et al., 2017; Larroya-García et al., 2018; Zhuang et al., 2018).

On the other hand, negative lifestyle aspects, among people living in our modern societies, are also considered important risk factors for the development of LOAD (van Praag, 2018). The most striking result of the epidemiological study above is that radical increases in Alzheimer's disease in Japan and substantial increase in developing countries are associated with changes in national diets (Grant, 2013). Furthermore, there are many undesirable lifestyle factors in the modern society that may contribute to AD development. These factors include unhealthy diet, lack of sleep, circadian rhythm disturbance, chronic noise, sedentary behavior etc., and, in turn, gut microbiome is highly sensitive to these factors. From this point of view, studying the links between modern lifestyle, gut microbiome and Alzheimer's disease is an important task that requires special attention. Understanding the interplays between the human microbiome and the brain, as well as the factors influencing these relations may contribute to a deeper understanding of AD etiology and may serve as a basis for the development of prophylactic measures to prevent or slow down the progression of the disease.

## Brain-Gut-Microbiota Axis and Alzheimer's Disease

In the past 10 years, considerable information has been accumulated on the action of microbiome on the central nervous system (CNS) and “brain-gut-microbiota axis” conception was proposed (Kowalski and Mulak, 2019). The CNS regulates the permeability, secretion, motility, and immunity of the digestive tract by exerting its effect on the enteric nervous system, muscle tissue and the mucous layer of the intestine through the efferent autonomic nervous pathways (Carabotti et al., 2015). In turn, the intestinal microbiome is able to influence brain functions through afferent signaling pathways and through the secretion of biologically active substances (Burokas et al., 2015; Petra et al., 2015). There is a number of published data showing the effects of intestinal dysbiosis, caused by changes in diet, the use of antibiotics, non-steroidal anti-inflammatory drugs as well as the presence of pathogenic microorganisms, on cognitive functions of the brain (Gareau, 2014; Jiang et al., 2017).

For example, acute stress and infection caused by conditional pathogenic bacteria *Citrobacter rodentium* have been shown to lead to memory disorders in C57BL/6 mice (Gareau et al., 2011). In sterile Swiss-Webster mice, bred in conditions precluding postnatal existence of bacteria in the intestine, a deficit of spatial and working memory was observed independent of infection and stress. This was accompanied by reduced neurotrophic brain factor (brain-derived neurotrophic factor, BDNF) expression (Gareau et al., 2011). BDNF is one of the key neurotrophins that play an important role in synaptic plasticity, and there is evidence of reduced BDNF levels in the brain and serum of patients with Alzheimer's disease (Michalski et al., 2015). On the contrary, studies conducted by Neufeld et al. revealed increased levels of BNDF in the central amygdale of sterile mice, reduced expression of mRNA encoding the serotonin receptor (5HT1A) and the NR2B subunit of the NMDA receptor (ionotropic glutamate receptor, selectively binding N-methyl-D-aspartate) in the dentate fascia of the hippocampus (Neufeld et al., 2011).

Wang et al. have demonstrated that in rats, intestinal dysbiosis caused by the usage of ampicillin for 1 month lowered the NMDA receptor and mineralocorticoid levels in the amygdala, increased the aggressiveness of the animals and caused impaired spatial memory while the presence of the *Lactobacillus fermentumNS9* strain in the intestinal microbiome normalized these parameters (Wang et al., 2015). Another study by Liang et al. showed that probiotic *Lactobacillus helveticusNS8* significantly improved cognitive impairment caused by chronic stress in Sprague-Dawley rats bred under sterile conditions (Liang et al., 2015). *L. helveticus* NS8 also reduced plasma levels of corticosterone and adrenocorticotropic hormone and increased the content of the anti-inflammatory cytokine IL-10, restored the level of serotonin and norepinifrine, and increased expression of BDNF in the hippocampus (Liang et al., 2015). Similar data were obtained by Luo et al. (2014) and Ohsawa et al. (2015). In addition, the probiotic *Bifidobacterium Longum 1714* improved cognitive function in male BALB/c mice (Savignac et al., 2015).

Studies of stool samples obtained from transgenic mice expressing the human APP gene and PS1 (CONVR-APPPS1, an animal model of Alzheimer's disease), showed significant differences in the composition of the intestinal microbiome of these animals compared to wildtype mice (Harach et al., 2017). In 8-month-old CONVR-APPPS1 mice there was a significant decrease in the number of *Firmicutes, Verrucomicrobia, Proteobacteria*, and *Actinobacteria*, and an increase in the content of bacteria belonging to the *Bacteroidetes* and *Tenericutes* compared to wild type mice of similar age. In addition, there was a significant decrease in Aβ deposits in the brain of CONVR-APPPS1 mice bred under sterile conditions compared to animals of the same genotype bred under standard conditions. Microbiota obtained from the intestines of CONVR-APPPS1 mice bred under normal conditions when introduced into intestines of mice bred under sterile conditions led to an increase in pathological Aβ deposits in the central nervous system, while fecal transplantation from wild-type mice did not lead to a significant increase in Aβ levels in the brain.

Studies carried out on laboratory animals are confirmed by clinical data obtained in the study of the intestinal microbiome of the elderly. The association of brain amyloidosis with pro-inflammatory intestinal bacterial taxa and peripheral markers of inflammation in people of old age suffering from cognitive disorders was shown (Cattaneo et al., 2016). The results of this study demonstrated that, in dementia patients with amyloidosis, an increased level of pro-inflammatory cytokines in the blood (IL-6, CXCL2, NLRP3, and IL-1β) was accompanied by a reduced content of *E. rectale* and an increased content of *Escherichia/Shigella* in stool samples. A positive correlation was also demonstrated between pro-inflammatory cytokines and the number of pro-inflammatory intestinal bacteria belonging to the *Escherichia/Shigella* taxon in stool samples, while a negative correlation was found between pro-inflammatory cytokines and the number of anti-inflammatory intestinal bacteria belonging to the *E. rectale* taxon.

A study of the composition of the intestinal microbiome in patients at the Alzheimer's Disease Research Center (Wisconsin Alzheimer's disease Research Center, USA) revealed significant differences in the composition of the intestinal microbiome in patients with AD and healthy people at the phylum and species levels (Vogt et al., 2017). These studies demonstrated a decrease in the number of bacteria in the *Firmicutes* and *Actinobacteria phyla* (in particular, bacteria of the genus *Bifidobacterium*), and an increase in the number of bacteria belonging to the *Bacteroidetes* and *Proteobacteria* phyla in the intestinal microbiome of AD patients. In general, quantitative differences were found between 13 genera of bacteria in AD patients and healthy study participants. In addition, a differential correlation was shown between the levels of individual bacterial genera in the intestine and cerebrospinal markers of AD, such as Aβ42/Aβ40, p-tau, as well as the Aβ/p-tau ratio (Vogt et al., 2017). Studies conducted at Chongqing Medical University (China) also revealed significant differences in the composition of bacteria present in the bowels of patients with AD in taxonomic groups such as *Bacteroides, Actinobacteria, Ruminococcus, Lachnospiraceae*, and *Selenomonadales* (Zhuang et al., 2018). However, qualitative changes in the intestinal microbiome in Chinese patients differed somewhat from those in the United States. Zhuang et al. showed a decrease in the number of bacteria belonging to the phylum *Bacteroidetes*, while the number of bacteria in the phylum *Firmicutes* remained unchanged compared with healthy controls. These differences may be related to a number of factors, including comorbidities, ethnicity, lifestyle, and dietary preferences (Tasnim et al., 2017).

Irrespective of bacterial taxa, the functional composition of the gut microbiota may also be important (Lozupone et al., 2012). In this regards, Liu et al. conducted function analysis of microbiome in AD patients, patients with amnestic mild cognitive impairment (aMCI) and healthy controls (HC) based on Kyoto Encyclopedia of Genes and Genomes (KEGG) functional pathway (Liu et al., 2019a). They identified 5 altered functional orthologs in AD patients using level 3 KEGG pathways. For instance, in AD there were enriched orthologs related to bacterial secretion system (membrane transport) and lipopolysaccharide biosynthesis (glycan biosynthesis and metabolism) compared to HC or aMCI subjects. In contrast, the orthologs related to N-Glycan biosynthesis and phenylalanine, tyrosine and tryptophan biosynthesis, and histidine metabolism in amino acid metabolism were reduced in AD patients, but enhanced in aMCI patients when compared to HC.

The results of these studies demonstrate that the changes in the taxonomic and functional composition of the intestinal flora are able to influence brain functions. Published data also provide an evidence of the effect of intestinal microbiome on the development of amyloid pathology and indicate the possible role of intestinal microbiome as one of the factors of AD pathogenesis. In turn, gut microbiome is a dynamic modifiable system highly sensitive to lifestyle and aging. Thus, in the subsequent chapters we discuss the modern lifestyle factors and aging-related gut microbiome influence and their relations to AD pathology.

## Gut Microbiome and Aging

Since advanced age is a major risk factor for AD, age-related physiological changes, including changes in the microbiome, may play a certain role in the development of dementia. In this regard, a number of studies have shown that the composition of the gut microbiome undergoes significant changes with age (Salazar et al., 2017; Nagpal et al., 2018). It was shown that general age-related changes in the composition of the intestinal microflora include an increase in the number of facultative anaerobes, changes in species dominance, while, at the same time, there is stability in the total number of anaerobes (Mariat et al., 2009; Satokari et al., 2010). Hopkins and co-authors observed that the levels of *Bifidobacterium* and *Lactobacillus* were lower in the group of elderly people compared to those of young individuals (Hopkins and Macfarlane, 2002). While the adult organism contains 4–5 species of the genus *Bifidobacterium*, only one of the dominant species of this genus is found in old age: *Bifidobacterium adolescentis*, or phenotypically close *Bifidobacterium angulatum* and *Bifidobacterium longum* (Gavini et al., 2001; Hopkins and Macfarlane, 2002). One of the possible explanations for the reduced number of species and quantitative composition of *Bifidobacteria* in the elderly people is the decrease in their adhesion to the intestinal wall due to changes in the chemical composition and structure of the colon mucous membrane, causing restricted functionality and immunological reactivity in the intestine as well as increased susceptibility to gastrointestinal infections (He et al., 2001). In turn, the bacteria *Bifidobacterium* and *Lactobacillus* are actively involved in the production of aminobutyric acid (γ-Aminobutyric acid, GABA) (Junges et al., 2018; Strandwitz, 2018). GABA is the most important inhibitory mediator of the central nervous system of humans and other mammals involved in neurotransmitter and metabolic processes in the brain. It has been proven that the level of aminobutyric acid in the intestine correlates with its level in the CNS. Decrease in the number of *Bifidobacterium* and *Lactobacillus* leads to brain dysfunction associated with synaptogenesis disorders, depression, and cognitive impairment (Strandwitz, 2018).

Many authors have noted that Bacteroides species diversity changes with age (Bartosch et al., 2004; Layton et al., 2006). In studies conducted by a group of scientists under the leadership of Tongeren, *Bacteroides/Prevotella, Eubacteriumrectale/Clostridium coccoides*, and *Ruminococcus* prevailed in the microbiota of people aged between 70 and 100 years (van Tongeren et al., 2005). The growth of proteolytic bacteria, such as *Fusobacteria, Propionibacteria*, and *Clostridia*, was shown in the intestinal microbiota of elderly people leading to the development of putrefactive processes, especially in patients with post antibiotic therapy. This is confirmed by data on the increase of proteolytic activity (Hopkins and Macfarlane, 2002; Woodmansey et al., 2004). Also, an increased number of pro-inflammatory enterobacteria, streptococci, staphylococci, and yeast cells were found which may be associated with an elevated level of serum antibodies to commensal (normal) intestinal microflora, such as *Escherichia coli* and *Enterococcus faecalis*.

## Unhealthy Nutrition Linked to Alzheimer's Disease and Gut Microbiome

So-called “Western diet” (WD), which is characterized by high intake of saturated fats and added sugars (Weisburger, 1997), is one of the symbols of the modern lifestyle and it is an established risk factor for AD development (Grant, 1999, 2016; Noble et al., 2017). For example, it has been demonstrated that AD rates increased from 1% in 1985 to 7% in 2008 in Japan, and this increase is associated with nutritional transition from the traditional Japanese diet to a Western diet (Dodge et al., 2012). In fact, preclinical experiments have confirmed that high fat diet (HFD) may change the gut microbiota and contribute to development of dementia (Studzinski et al., 2009; Nam et al., 2017; Sah et al., 2017; Sanguinetti et al., 2018). These studies demonstrated that HFD promoted cognitive impairment by inducing oxidative stress and deteriorative neuronal apoptosis via inactivation of Nrf2 signaling pathway (Studzinski et al., 2009; Nam et al., 2017; Sah et al., 2017; Sanguinetti et al., 2018). Nam and coauthors showed that HFD significantly increased amyloid deposition and reduced cognition of 12-months old APP23 mice (Nam et al., 2017). In this study, RNA-seq results showed that genes related to immune response, such as Trem2 and Tyrobp in HFD mice were upregulated, but expression of the genes related to neuron projections and synaptic transmission was decreased. The authors demonstrated that levels of 24 lipid sub-species in the brain were significantly modulated by HFD.

In turn, recent study of microbiome-metabolome signatures in 3xTg-AD mice genetically predisposed to AD and fed a normal or fatty diet have demonstrated that high-fat feeding and genetic predisposition to neurodegenerative disease share common abnormalities in the gut microbiome (Sanguinetti et al., 2018). The authors showed that HFD changed bacterial composition in both colon and caecum, and also lead to reduced abundance of the microorganisms compared to normal diet-fed animals (ND). In this study, HFD mice had elevated abundances of *Firmicutes* than *Bacteroidetes* at phylum levels, *Rikenellaceae, Lachnospiraceae, Enterococcaceaeand* S24.7 at family level, as well as elevated amount of fecal ribose. High level of *Clostridium* and *Staphylococcus* were also found in the caecum. Study of serum and fecal metabolites revealed a deficiency in unsaturated fatty acids and choline, and an excess in ketone bodies, lactate, amino acids, TMA, and TMAO in 3xTg-AD mice fed a fatty diet. These metabolic changes were associated with high abundance of *Enterococcaceae, Staphylococcus, Roseburia, Coprobacillus*, and *Dorea*, and a low level of *Bifidobacterium*, which in turn are related to cognitive impairment and cerebral hypometabolism.

## Sedentary Behavior Linked to Alzheimer's Disease and Gut Microbiome

Sedentary lifestyle is becoming a significant public health issue in many countries despite being linked to a number of chronic health conditions (Owen et al., 2010). Accumulating evidence indicates that sedentary behavior can be a risk factor for cognitive decline (Wheeler et al., 2017), while physical exercise may be an effective strategy for preventing dementia (Fenesi et al., 2016). It has been shown that the mechanisms underlying the neuroprotective influence of physical activity on Alzheimer's disease are: the production of antioxidant enzymes and growth factors and decrease in ROS and neuroinflammation, the concentration of Aβ plaques and tau protein in the brain (Chen et al., 2016b).

There is also data indicating that exercise can influence gut microbiome (Fernandez et al., 2018), and this effect is especially prominent in obese people and sedentary women (Allen et al., 2017; Bressa et al., 2017). In addition, recent data has demonstrated that physical exercise and probiotics are able to reduce the levels of Aß in the brain and slow down progression of AD symptoms in transgenic APP/PS1TG mice (Abraham et al., 2019). The authors demonstrated that physical training was capable of increasing abundance of butyrate-producing bacteria i.e., *Butyrivibrioproteoclasticus* and *Marvinbryantiaformatexigens*, and reducing pro-inflammatory bacteria, such as Clostridium, *Eubacterium*, and *Roseburia*. Moreover, exercise decreased the levels of H_2_O_2_ generating bacteria *L. johnsonii*. It has been suggested that butyrate is a key regulator of inflammatory processes that induces mucin production and reduces the penetration of LPS from intestine into the bloodstream. The authors also concluded that physical activity or proper nutrition alone has a weak effect on dementia, whereas together their effects become significant.

## Sleep Deprivation Linked to Alzheimer's Disease and Intestinal Microbiome

Insufficient sleep is another important public health issue of the twenty-first century (Chattu et al., 2018), and it has been suggested that disrupted sleep may promote the development of Alzheimer's disease (Ju et al., 2013a,b; Shokri-Kojori et al., 2018). For example, animal studies have demonstrated that acute sleep deprivation significantly increases Aβ levels in brain interstitial fluid (Kang et al., 2009), and, in contrast, natural, and anesthetic sleep increases the interstitial space and subsequently enhances convective exchange of cerebrospinal fluid with interstitial fluid resulting in increased rate of Aβ clearance (Xie et al., 2013). Clinical studies have also shown that healthy individuals have morning decrease in Aβ42 in cerebrospinal fluid, but 24 h of total sleep deprivation undoes this decrease (Ooms et al., 2014). Similarly, it has been found that slow wave sleep disruption correlates with an increase in Aβ40 level in cerebrospinal fluid (Ju et al., 2017) and that one night of sleep deprivation induces Aβ accumulation in the brains of healthy individuals (Shokri-Kojori et al., 2018).

It is of our interest that chronic sleep disruption impacts gut microbiome. A study in animals has revealed that chronic sleep fragmentation alters taxonomic profiles of fecal microbiota and induces systemic and adipose tissue inflammation and insulin resistance (Poroyko et al., 2016). Similarly, randomized within-subject crossover study conducted by Benedict et al. demonstrated that partial sleep deprivation (PSD) in normal-weight young individuals affects the human gut microbiota. In particular, PSD increased *Firmicutes:Bacteroidetes* ratio with a higher abundance of the families *Coriobacteriaceae* and *Erysipelotrichaceae*, and lower abundance of *Tenericutes* (Benedict et al., 2016). Contrary to this, Zhang at al. reported that major microbial populations were not altered in sleep-restricted rats and healthy human subjects (Zhang et al., 2017). The authors concluded that the microbiome is largely resistant to changes during sleep restriction and that sleep disruption and microbial dysbiosis are independent health risk factors (Zhang et al., 2017). However, in another research, better sleep quality in healthy older adults was associated with better neuropsychological test performance and higher abundance of microbial phyla *Verrucomicrobia* and *Lentisphaerae* in the stool samples (Anderson et al., 2017). Collectively, these studies suggest that a lack of sleep combined with obesity, diabetes and high-fat diet can be a risk factor for Alzheimer's disease and is associated with changes in the gut microbiome.

## Circadian Rhythms, Intestinal Microbiome and Alzheimer's Disease

A phenomena known as “social jetlag,” or the mismatch between social and biological clocks, is common in the modern society and causes circadian rhythm disruption (CRD) (Farhud and Aryan, 2018). One of the causes of CRD is light pollution, which is a typical hallmark of the big cities (Chepesiuk, 2009). It is a matter of fact that sleep deprivation and CRD is one of the common and earliest signs of AD, and there is increasing evidence that CRD might be a contributing factor in AD pathogenesis (Wu et al., 2003; Musiek, 2015; Musiek and Holtzman, 2016; Phan and Malkani, 2019). In support of this notion, there is a study demonstrating that Aβ production is regulated by circadian rhythms with peak concentrations of Aβ occurring during wakefulness (Kang et al., 2009), which is in agreement with the data discussed in the previous chapter. A recent study has shown that targeted deletion of the core clock gene *Bmal1* in *APPPS1-21* transgenic mice resulted in disruption of daily hippocampal interstitial fluid Aβ oscillations, increased expression of *Apoe* and promoted amyloid plaque accumulation (Kress et al., 2018). In addition, it has been demonstrated that the level of melatonin, one of the important regulators of circadian system, is reduced in AD patients (Wu et al., 2003). There is data indicating that melatonin and the circadian rhythms regulate the intestinal microbial flora (Zhu et al., 2018; Parkar et al., 2019), and that circadian rhythm disruption by abnormal light–dark (LD) cycles results in the dysfunction of the intestinal barrier and increases the number *Ruminococcus torques* but reduces that of *Lactobacillus johnsonii* (Deaver et al., 2018). In addition, circadian disruption affects functional gene composition of gut microbiome leading to downregulation of the genes involved in promoting host beneficial immune responses and upregulation of the genes involved in the synthesis and transportation of lipopolysaccharides (LPS) (Deaver et al., 2018), which is similar to the changes of the functional composition of the gut microbiome in AD patients (Liu et al., 2019a).

## Chronic Noise Stress, Intestinal Microbiome and Alzheimer's Disease

A large number of sources of noise pollution has appeared in the human environment since the onset of post-industrial era (Passchier-Vermeer and Passchier, 2000), thus making chronic noise another hallmark of the modern lifestyle (Seidman and Standring, 2010). Epidemiological and experimental studies showed that chronic noise has been associated with cardiovascular diseases, hearing impairment, changes in the immune system and birth defects (Passchier-Vermeer and Passchier, 2000; Ising and Kruppa, 2004). Recently, an etiological association between chronic noise exposure and Alzheimer disease was proposed (Cui and Li, 2013). In 2018, a retrospective cohort study conducted by Carey and coauthors demonstrated positive association between residential levels of noise and air pollution across London and incidence of dementia (Carey et al., 2018). A number of animal studies have shown that chronic noise induces tau pathology in the hippocampus and the prefrontal cortex (Manikandan et al., 2006; Cui et al., 2009, 2012a,b). Also, there is published data demonstrating upregulated expression of amyloid precursor protein (APP) and its cleavage enzymes, β- and γ-secretases upon chronic noise exposure (Cui et al., 2015), and the involvement of the corticotropin-releasing factor system in noise-induced alteration in Aβ production (Gai et al., 2017). Furthermore, Cui and et al. reported that cognitive impairment and Aβ accumulation in exposed-to-chronic-noise young SAMP8 mice was associated with the dysregulation of intestinal microbiota (Cui et al., 2018). The authors also found downregulation of endothelial tight junction proteins both in the intestine and brain and upregulation of serum neurotransmitter and inflammatory mediator levels in the mice. Further analysis of gut microbiota has revealed increased abundance of *Firmicutes* and reduced quantity of *Bacteroidetes* on the phylum level, whereas *Candidatus Jettenia, Denitratisoma*, and *SM1A02* levels were increased in noise exposed SAMP8 mice (the genus level) (Cui et al., 2018). Taken together, the studies suggest that chronic noise affects gut microbiome and can be one of the AD triggers.

## Possible Mechanisms Underlying the Effect of Gut Microbiome on the Pathogenesis of Alzheimer's Disease

Reducing the number and species diversity of many beneficial anaerobes such as *Bifidobacterium* and *Lactobacillus*, as well as a shift in the diversity of the intestinal microbiota toward conditional pathogenic and pathogenic microorganisms, results in changes in local intestinal chemical and immunological parameters and induces the translocation of the gut bacteria into focal lymphoid tissue (Nagpal et al., 2018). These factors contribute to an increase in permeability of the intestinal and blood-brain barriers and the penetration of pathological microflora and their metabolites into the brain (Tran and Greenwood-Van Meerveld, 2013; Elahy et al., 2015).

On the other hand, intestinal bacteria are able to excrete functional amyloid peptides and lipopolysaccharides (LPS) in large quantities. Amyloid peptide in bacteria contributes to various physiological processes on the surface of bacterial cells, such as biofilm formation, adhesion, interaction with other bacterial and eukaryotic cells, etc. Its structure and biophysical properties are similar to human pathological amyloid (Evans et al., 2018). For example, pro-inflammatory conditional pathogenic bacteria of the intestine such as *Escherichia coli, Baccilus subtilis, Salmonella tyrhimurium*, and *Salmonella enterica* are able to secrete large amounts of the bacterial amyloid peptide *curli* (Hufnagel et al., 2013; Schwartz and Boles, 2013). The *curli* peptide, like Aβ, forms a secondary structure of β-folded sheets and stains with thioflavin and Congo red (dyes used to stain the brain's amyloid plaques). It was shown that the main structural subunit of the *curli* peptide, the precursor of amyloid gA (gA amyloid precursor), has in its structure sections similar to Aβ42, and that sections can be recognized by the human TLR2 receptor (tall-like receptor 2) (Rapsinski et al., 2015). Interaction of TLR2 with *curli* peptide or human Aβ42 leads to the activation of bone marrow macrophages and their production of pro-inflammatory cytokines, such as IL-6 and IL-1β (Rapsinski et al., 2015). In a similar study, microbial amyloid was shown to be able to activate T-lymphocytes and induce the production of pro-inflammatory interleukins IL-17A and IL-22(Nishimori et al., 2012). These cytokines are able to penetrate the blood-brain barrier and cause the production of reactive oxygen species, activation of the TLR2/1 and NFB signaling pathways in microglia and astrocytes, which is directly related to neuroinflammation and neurodegeneration (Perriard et al., 2015; Sun et al., 2015; Zhan et al., 2018). Besides Chen et al. demonstrated that oral contamination of old rats (previously subjected to antibiotic treatment) with wild *E. coli* strain, capable of producing the functional curli peptide, led to an increase in brain tissue microgliosis and astrogliosis and increased expression of TLR2, IL6, and TNF (Chen et al., 2016a).

In addition to the amyloid peptide, many intestinal bacteria secrete LPS. LPS are the main components of the outer cell wall of gram-negative bacteria and, in the case of penetration from the intestinal cavity into the bloodstream, can cause neuro-inflammatory reactions. Published data indicates that the LPS level in the blood plasma of patients suffering from sporadic lateral sclerosis and AD is three times higher than the physiological age norm (Zhang et al., 2009). Post-mortem studies revealed that the level of LPS in the neocortex and hippocampus in patients with AD was two to three times (and in some cases 26 times) higher than in older people of the same age who did not suffer from cognitive disorders (Zhao et al., 2017). Studies on laboratory animals showed that intraventricular administration of LPS for 4 weeks can cause chronic neuroinflammation, nerve cell death of II and III layers of the entorhinal cortex and impairment of the long-term synaptic plasticity of the neurons of the dentate gyrus of the hippocampus, which is one of the characteristic signs of damage to the temporal lobes of cerebral hemispheres inAD (Hauss-Wegrzyniak et al., 2002).

There is also evidence that LPS secreted by bacteria *Bacteroidesfragilis* can activate the pro-inflammatory transcription factor NFB involved in the pathogenesis of AD in human primary microglial cells (Zhao and Lukiw, 2018). NFB induces transcription of pro-inflammatory miRNAs, such as miRNA-9, miRNA-34a, miRNA-125b, miRNA-146a, and miRNA-155, activating neuro-inflammatory mediators and inhibiting phagocytosis (Zhao and Lukiw, 2018). For example, micro-RNA-34a has been shown to inhibit TREM2 expression (the triggering receptor expressed on microglia/myeloid cells-2), thereby disrupting the phagocytic ability of microglia and increasing Aβ42 accumulation (Bhattacharjee et al., 2016). In support of this concept, the intraperitoneal administration of LPS to mice line C57BL/6J led to an increase in Aβ42 level in the brain and induced cognitive deficits (Kahn et al., 2012). *In vitro*, endotoxins secreted by *Escherichia coli* strains (*E. coli*) accelerated Aβ aggregation and fibril formation (Asti and Gioglio, 2014). Jaeger and colleagues showed that intraperitoneal administration of LPS disrupts Aβ transport through the blood-brain barrier, increasing its influx and decreasing efflux (Jaeger et al., 2009). It was also shown that intraventricular infusion of LPS in combination with ascorbic acid increased the immunoreactivity of intra-neuronal beta-amyloid (Hauss-Wegrzyniak and Wenk, 2002).

**Figure 1:**
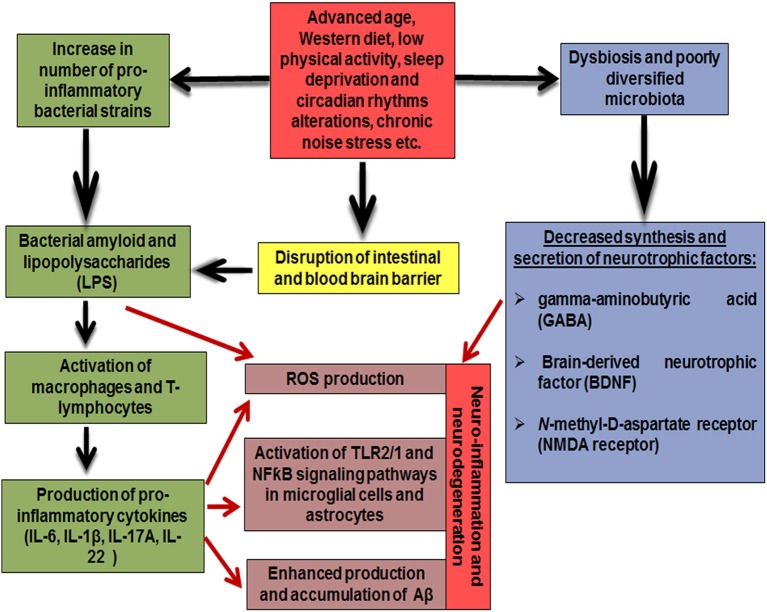
Mechanisms underlying the effect of gut microbiome on the pathogenesis of Alzheimer's disease.

Thus, the composition of gut microbiome changes significantly with aging: the diversity of beneficial bacteria, such as *Lactobacillus and Bifidobacteria*, decreases, and, in a contrast, a number of “unhealthy” pro-inflammatory bacteria, such as *Propionibacteria, Fusobacteria, Shigella*, and *Clostridia* increases. Furthermore, unhealthy modern lifestyle factors including high fat diet, sedentary behavior, lack of sleep, circadian rhythm disturbance and chronic noise alter the composition of gut microbiome in the same way, thus, exacerbating negative impact of aging. In turn, lack of probiotic strains affects synthesis and secretion of the neurotrophic factors, such as BDNF, NMDA receptor and GABA, while pro-inflammatory gut microbiota taxa are capable of secreting bacterial amyloid and lipopolysaccharides, which are considered to be neurotoxic. Since elderly people experience impaired barrier functions of the intestinal wall and the blood-brain barrier, these endotoxins are able to penetrate from the intestinal cavity into the bloodstream, and further into the brain tissue, and have a direct and/or systemic negative effect on the structure and functions of the CNS and promote the development of neuro-inflammation and neurodegeneration (Figure 1).

## Oral Microbiome and Alzheimer's Disease

There is new scientific evidence published recently that aside the gut microbiome, oral microflora is also able to influence brain functions (Orr et al., 2020). Numerous studies have shown that periodontal disease is associated with neurodegeneration and cognitive decline (Kamer et al., 2008; Cerajewska et al., 2016; Wang et al., 2019). Chang and coauthors reported that chronic periodontitis of 10 years duration was associated with a 1.707-fold increase in the risk of developing AD (Chen et al., 2017). A nationwide, retrospective, matched-cohort study in Taiwan showed that patients with chronic periodontitis and gingivitis have a higher risk of developing dementia compared to those with healthy gums (Tzeng et al., 2016). Moreover, recent accumulating evidence has demonstrated a causal relationship between oral microbiome and AD (Paganini-Hill et al., 2012; Harding et al., 2017; Liu et al., 2019b; Panza et al., 2019; Olsen and Singhrao, 2020). For example, *P. gingivalis*, the most common periodontal bacteria causing periodontal disease, was capable of inducing accumulation of amyloid-beta plaques and neurofibrillary tangles following experimental oral infection in mice (Dominy et al., 2019). In turn, serum antibodies for *P. gingivalis* have been found to be elevated in AD patients (Kamer et al., 2009), and protein-degrading enzyme gingipain produced by *P. gingivalis*, was found in the brain of Alzheimer's patients (Singhrao and Olsen, 2019). Dominy et al. have demonstrated that oral administration of small-molecule inhibitors of gingipain block gingipain-induced neurodegeneration, decreased *P. gingivalis* load in the mouse brain and the host Aβ42 response *to P. gingivalis* brain infection (Dominy et al., 2019). It was also found that chronic systemic *P. gingivalis* infection causes Aβ accumulation in inflammatory monocytes/macrophages via the activation of CatB/NF-κB signaling (Kunkle et al., 2019).

In addition, there is data indicating the significant differences in quantity and quality of oral microbiome in AD patients compared to mentally healthy individuals of the same age. For example, Liu et al. have demonstrated lower richness and diversity of salivary microbiome in patients with Alzheimer's disease compared to healthy controls (Liu et al., 2019b). The authors reported a relatively high level of *Moraxella, Leptotrichia*, and *Sphaerochaeta* and significantly decreased number of *Rothia* in the saliva of AD patients (Liu et al., 2019b). However, these authors emphasize the limitations of the study due to the absence of many periodontal bacteria in saliva which exist within the subgingival niche or dental plaques (Filoche et al., 2009). Therefore, a comprehensive picture of the full composition of oral microbiome in patients with AD requires further research.

## Conclusion

Recent studies strongly suggest that gut and oral microbiome is capable of modulating the neurochemical and neuro-metabolic signaling pathways of the brain through the formation of a two-way communication axis involving the endocrine and immune systems, and contribute to the development of neuro-inflammation and neurodegeneration. In turn, there is a strong correlation that exists between AD and modern lifestyle factors. It is a fact that unhealthy diet, lack of sleep, circadian rhythm disturbance, chronic noise, and sedentary behavior are linked to neurodegeneration. By focusing on the mechanism for interaction between lifestyle factors and AD, we can evaluate the contribution of changing modern society to the increase in prevalence of AD. However, tackling this issue is impossible without understanding its intertwined relationship with other aspects, and gut microbiome is crucial for this interaction. From this point of view, the study of the composition of the intestinal microbiome in patients with AD and healthy aging people is of considerable interest. This could unearth novel associations between intestinal microbiome, lifestyle, and dementia, and help to develop practical recommendations for the prevention and treatment of this severe pathology.

## Author Contributions

SA conceptualized the structure, wrote the first draft, and edited the final version of the manuscript. BU co-wrote the first draft. A-RM, AKa, YS, AT, FO, and AKu made substantial contribution to the content. All authors approved the final version of the manuscript.

### Conflict of Interest

The authors declare that the research was conducted in the absence of any commercial or financial relationships that could be construed as a potential conflict of interest.
